# The cat as a small dog?—Comparison of trabecular and cortical bone microarchitecture of radius and ulna in cats and small dogs using microcomputed tomography

**DOI:** 10.1002/vms3.619

**Published:** 2021-08-27

**Authors:** Franziska Planner, Franziska Feichtner, Andrea Meyer‐Lindenberg

**Affiliations:** ^1^ Clinic for Small Animal Surgery and Reproduction Ludwig‐Maximilians‐University Munich Germany

**Keywords:** canine, distal radius fracture, feline, microcomputed tomography, morphometrical analysis

## Abstract

The forearms of dogs and cats do not only differ anatomically from each other, but there are also differences in prevalence of radius and ulna fractures between the two species. The prevalence of antebrachial fractures is 18.0% in dogs and 2.0–8.0% in cats. Many studies focus solely on the trabecular and cortical bone structure of dogs and the characteristics of the cat are often disregarded.

The aim of this study was to evaluate the trabecular structure parameters [bone volume fraction per total volume (BV/TV), bone surface per total volume (BS/BV), trabecular number (Tb.N), trabecular thickness (Tb.Th), trabecular separation (Tb.Sp), connectivity density (Conn. D), degree of anisotropy (DA)] and the diaphyseal cortical bone density (Mean Density) of the antebrachium in cats and small dogs to visualise their differences.

For this purpose, a total of 32 forearms of cats (*n* = 8) and small dogs (*n* = 8) were evaluated using microcomputed tomography and the findings were compared.

The results of the study showed that cats had higher values for BV/TV, Tb.Th, Tb.Sp, DA and Mean Density and lower values for BS/BV, Tb.N and Conn.D at radius and ulna compared to dogs.

According to the results of this study, the higher bone volume fraction (BV/TV), thicker trabeculae (Tb.Th), increased anisotropy (DA) and significantly higher diaphyseal cortical density (Mean Density) could contribute to the lower fracture risk of the antebrachium in cats compared to small dogs.

## Graphical abstract

In the present study trabecular and cortical bone structure of the antebrachium of cats and small dogs were investigated and the differences had been visualized. Comparative studies of feline and canine structural bone architecture are not found at all in the current literature. Furthermore the aim of this study was to detect reasons for the decreased antebrachial fracture incidence of cats comparing to dogs

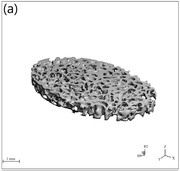

## 1 INTRODUCTION

In some veterinary studies, the term carnivores is often used, with no distinction made between dogs and cats. Many anatomical and clinical studies focus solely on dogs (Scott & McLaughlin, 2008). The characteristic features of the cats are disregarded and they are often treated as small dogs (Scott & McLaughlin, 2008).

Anatomical peculiarities of cats compared to dogs can also be verified on the antebrachial bones (Chandler & Beale, 2002). The feline round *Fovea capitis radii* differs from the broader, dorsally retracted fovea of the dog. The *Tuberositas radii* can only be recognised as a rough elevation in the dog. The radial shaft, *Corpus radii*, is almost smooth in cats, but in dogs the contact surface to the ulna is rough (Nickel et al., 1987). Proximal to the *Facies articularis carpea* is the *Crista transversa*, which unfolds as a prominent groin in cats and a transverse bulge in dogs (Nickel et al., 1987). The *Ulna* is distally more tapered in the dog than in the cat. The *Tuber olecrani* appears triangular in the dog and round in the cat. The *Incisura trochlearis* is divided by a sagittal crest into a larger lateral and a smaller medial part only in dogs (König & Liebich, 2001; Nickel et al., 1987; Vollmerhaus et al., 1994).

In addition, the two animal species differ in the movement of the radioulnar joint. Only the cat is able to actively perform considerable supination movements. In dogs, the rotation of the forearm can only be passive (Roos et al., 1992).

Differences between the two species can also be established within radius and ulna fractures. In cats, these can be recorded with a prevalence of 2.0–8.0% (Harari, 2002). In dogs, antebrachial fractures are the third most common limb fractures, with a prevalence of 18.0% (Boudrieau, 2003; Harasen, 2003).

The main causes of forearm fractures in dogs and cats are falls from great heights and road traffic accidents (Harasen, 2003; Meyer, 1977; Wetscher, 2012). Further causes in both species are other direct traumatic events, such as bite wounds, getting stuck, kicks and entrapment (Harasen, 2003; Meyer, 1977; Wetscher, 2012).

While various studies have focused on trabecular structural analysis at different locations in dogs (Bagi et al., 2011; Fitzpatrick et al., 2016; Hu et al., 2002; Lau et al., 2013; Scherzer et al., 2009), studies on trabecular bone formation in feline bones are very limited (Boyd et al., 2005). Comparative studies of feline and canine structural bone architecture are not found in the current literature.

Therefore, in the present study the trabecular structural parameters and diaphyseal cortical bone density of the antebrachium of cats and small dogs are assessed by microcomputed tomography and the differences between these animal species are presented, with regard to the different fracture prevalence.

## 2 MATERIALS AND METHOD

### 2.1 Experimental model

The experiment was approved by the Animal Ethics committee of the Faculty of Veterinary Medicine, Ludwig‐Maximilians‐University, Munich, Germany.

The forearm bones used in this study were obtained from dogs and cats that died or were euthanised for various reasons. Diseases of the musculoskeletal system that could possibly influence the study were radiologically excluded. The carcasses were frozen at –21°C after euthanasia of the animals. For examination the animals were thawed at room temperature. Radius and ulna were disarticulated at the elbow joint and the skin, surrounding muscles and ligaments were dissected down to the carpus. The antebrachial bones were preserved in 4.0% formalin for a maximum of 1 week until further microcomputed tomographic measurements.

### 2.2 Microcomputed tomographic measurements

For the microcomputed tomographic scans, the forearm bones were placed on a self‐made polystyrene piece with the help of a specially manufactured plastic half‐shell in the gantry of the device and fixed with adhesive tape, with the palmar side of the antebrachii facing upwards.

The samples were then scanned by microcomputed tomography (XtremeCT II: Scanco Medical, Zurich, Switzerland). The scan area included radius, ulna and the carpal bones. For this study, a tube voltage of 68 kV, a voxel size of 30.3 μm, an integration time of 200 ms and 1000 projections/180° were chosen.

### 2.3 Microcomputed tomographic evaluation

The μCT Evaluation Program V6.6 (Scanco Medical, Zurich, Switzerland) was used to evaluate the trabecular and cortical bone structure of radius and ulna.

**FIGURE 1:**
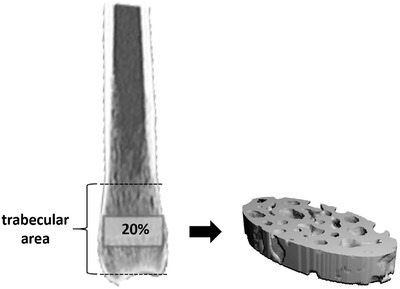
Presentation of the cylindrical Region of Interest (ROI) for the measurement of trabecular bone structure, exemplarily shown in the distal radius of a cat. First, the trabecular region was visually limited so that at the beginning and end at least 50.0% of the trabecular network was still visible in the bone cross‐section (trabecular area). Subsequently, the mean 20.0% was determined as the length for the cylindrical ROI

**FIGURE 2:**
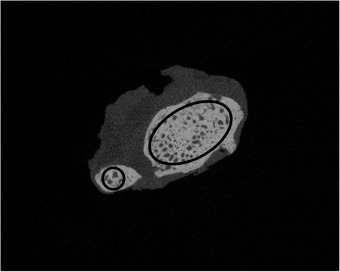
Presentation of the diameter of the cylindrical Regions of Interest (ROIs) in the distal trabecular bone of a cat. A circle at the ulna or an oval at the radius was adapted, which included as much cancellous bone as possible in diameter without including the cortex

Trabecular bone can be located in the proximal and distal epi‐ and metaphysis of the radius and ulna. At these four locations, proximal ulna, proximal radius, distal ulna, and distal radius, the mean 20.0% of the entire cancellous area was limited and defined as cylindrical Regions of Interest (ROIs) (Figure 1). The beginning and the end of the cancellous region were chosen so that at least 50.0% of trabecular structures were visible in the cross‐section of the bone. The diameter of the cylindrical ROIs was adjusted, as shown in Figure 2, to include as much cancellous bone as possible, but not the cortex.

**FIGURE 3:**
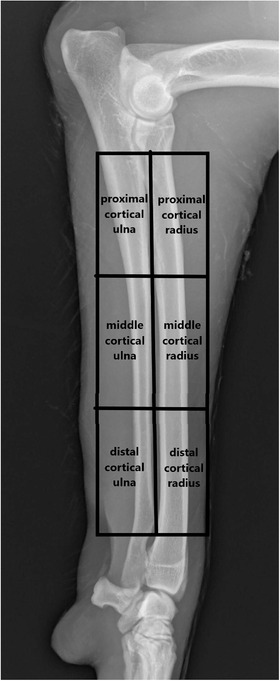
Presentation of the cylindrical Regions of Interest (ROIs) for measurement in diaphyseal bone: proximal cortical ulna, proximal cortical radius, middle cortical ulna, middle cortical radius, distal cortical ulna and distal cortical radius. Each ROI corresponds to one third of the diaphysis of the bones

In addition, the entire diaphysis was evaluated for cortical density. The beginning and the end were defined in such a way that a maximum of three trabecular connections were still visible in the cross‐section of the bone in the medullary cavity. Finally, the diaphysis was divided into three parts: distal, middle and proximal third, where cylindrical ROIs were located (Figure 3).

Thresholds for cancellous bone and diaphyseal cortex were determined at each location for each group by two independent observers using a mean value. The thresholds were used to evaluate trabecular structural parameters [bone volume fraction BV/TV (%), bone surface to volume ratio of bone BS/BV (mm^–1^), trabecular number Tb.N (mm^–1^), trabecular thickness Tb.Th (mm), trabecular separation Tb.Sp (mm), Connectivity‐Density Conn. D (mm^–3^), Degree of Anisotropy DA] and the diaphyseal Mean Density (mg HA/ccm).

### 2.4 Statistics

The statistical analysis and graphic presentation were performed using IBM SPSS Statistic 26.0 (IBM Corp., Armonk, NY, USA).

First, a Shapiro–Wilk and Kolmogorov–Smirnov tests were performed to test for normal distribution.

Subsequently, all values of the descriptive statistics were determined. The normally distributed data of the two groups were compared for each structure parameter and each location using a *t*‐test. For non‐normally distributed data, a Mann–Whitney *U*‐test was applied.

Significant differences were assumed from a probability of error of *p* < 0.05.

## 3 RESULTS

For the aim of the present study, a total of 32 forearms of deceased or euthanised cats and dogs were examined by microcomputed tomography. The different animal species were divided into two groups. One group contained eight cats from 1.7 to 6.2 kg, including five European Shorthair, one European Longhair, one British Shorthair and one Maine Coon. The second group contained three Poodles, two Terrier mongrels, one Terrier‐Dachshund mongrel, one Dachshund mongrel and one Dachshund weighing 5.8–10.0 kg. In both groups, only adult animals were included and the gender distribution was balanced in order to avoid differences due to age, sex or neutering.

When comparing the mean values from the descriptive statistics of the bone volume fraction (BV/TV) in the trabecular bone of dogs and cats, it became apparent that the cats showed higher mean values than the dogs at all four locations (Table 1).

**TABLE 1:** Mean values and standard deviation of bone parameters per localisation and group

		**Dogs**	**Cats**
**BV/TV (%)**	**Distal ulna**	36.98 ± 9.62	45.79 ± 20.95
	**Distal radius**	39.65 ± 8.53	42.86 ± 14.12
	**Proximal ulna**	37.30 ± 6.42	40.85 ± 17.76
	**Proximal radius**	43.54 ± 6.37	44.41 ± 3.52
**BS/BV (mm^–1^)**	**Distal ulna** *	15.10 ± 3.28	9.79 ± 4.46
	**Distal radius**	13.37 ± 2.30	9.76 ± 5.36
	**Proximal ulna** *	12.21 ± 2.53	9.91 ± 5.10
	**Proximal radius** *	11.58 ± 1.70	9.30 ± 3.95
**Tb.N (mm^–1^)**	**Distal ulna** *	2.65 ± 0.17	1.85 ± 0.30
	**Distal radius** *	2.33 ± 0.23	1.88 ± 0.63
	**Proximal ulna** *	2.52 ± 0.26	1.90 ± 0.46
	**Proximal radius** *	2.48 ± 0.22	1.81 ± 0.38
**Tb.Th (mm)**	**Distal ulna** *	0.14 ± 0.03	0.25 ± 0.12
	**Distal radius**	0.17 ± 0.04	0.31 ± 0.36
	**Proximal ulna** *	0.15 ± 0.02	0.21 ± 0.07
	**Proximal radius**	0.18 ± 0.02	0.26 ± 0.13
**Tb.Sp (mm)**	**Distal ulna**	0.24 ± 0.05	0.31 ± 0.16
	**Distal radius**	0.26 ± 0.05	0.39 ± 0.30
	**Proximal ulna**	0.25 ± 0.05	0.36 ± 0.23
	**Proximal radius** *	0.23 ± 0.04	0.31 ± 0.08
**Conn.D (mm^–3^)**	**Distal ulna** *	20.73 ± 5.66	6.75 ± 4.06
	**Distal radius** *	11.90 ± 3.39	8.14 ± 6.23
	**Proximal ulna** *	17.19 ± 4.01	6.23 ± 3.53
	**Proximal radius** *	15.37 ± 3.27	7.901± 5.90
**DA**	**Distal ulna** *	1.61 ± 0.24	1.84 ± 0.23
	**Distal radius**	1.83 ± 0.22	2.13 ± 0.57
	**Proximal ulna** *	1.33 ± 0.09	1.98 ± 0.33
	**Proximal radius** *	1.69 ± 0.07	2.16 ± 0.39
**Mean density (mg HA/ccm)**	**Distal cortical ulna** *	1150.53 ± 38.16	1226.48 ± 74.71
	**Middle cortical ulna** *	1182.69 ± 29.41	1249.11 ± 68.07
	**Proximal cortical ulna** *	1190.40 ± 22.23	1259.34 ± 58.64
	**Distal cortical radius** *	1211.12 ± 27.50	1250.19 ± 67.98
	**Middle cortical radius** *	1208.79 ± 39.56	1269.69 ± 58.64
	**Proximal cortical radius** *	1200.60 ± 28.75	1272.90 ± 59.90

* *p* < 0.05.

**FIGURE 4:**
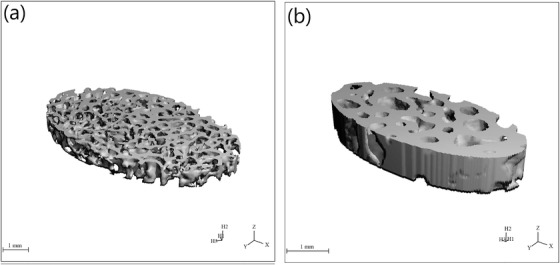
Trabecular bone architecture at the distal radius of a representative dog (a) and a representative cat (b). The dog shows significantly more and thinner trabeculae compared to the cat

An opposite pattern was observed when comparing the ratio of trabecular bone surface area (BS) to trabecular bone volume (BV). For BS/BV lower mean values were observed at all locations for cats compared to small dogs, with *p*‐values of 0.001 at the distal ulna, 0.003 at the proximal ulna and 0.042 at the proximal radius. The cats had fewer and thicker trabeculae (Tb.N, Tb.Th) with greater trabecular separation (Tb.Sp) than dogs at all locations (Table 1 and Figure 4a, b). These results were obtained for Tb.N at the distal ulna (*p* = 0.000), distal radius (*p* = 0.026), proximal ulna (*p* = 0.000) and proximal radius (*p* = 0.000), for Tb.Th at the distal (*p* = 0.001) and proximal ulna (*p* = 0.008) and for Tb.Sp at the proximal radius (*p* = 0.001).

The connectivity (Conn.D) of trabecular bone was lower in cats than in dogs (Table 1). This was confirmed at all locations (distal ulna with *p* = 0.000, distal radius with *p* = 0.042, proximal ulna with *p* = 0.000, proximal radius with *p* = 0.001).

When comparing the anisotropy (DA) of the trabecular bones, higher values were found in cats than in dogs at all locations (Table 1), with *p* = 0.013 at the distal ulna, *p* = 0.000 at the proximal ulna and *p* = 0.000 at the proximal radius.

The two groups showed distinctions in the Mean Density of the diaphysis (Table 1). In the cats, a denser cortex was visible in all six investigated locations compared to the dogs (distal cortical ulna with *p* = 0.001, middle cortical ulna with *p* = 0.024, proximal cortical ulna with *p* = 0.000, distal cortical radius with *p* = 0.000, middle cortical radius with *p* = 0.001 and proximal cortical radius with *p* = 0.000).

## 4 DISCUSSION

In the present study, cortical bone density and trabecular microarchitecture of the radius and ulna in dogs and cats were examined comparatively.

It was possible to show the cortical and trabecular differences between the canine and feline antebrachium, as well as possible reasons for the clearly lower prevalence of radius and ulna fractures in cats (Boudrieau, 2003; Harari, 2002; Harasen, 2003; Nolte et al., 2005). The risk of fracture is determined by bone density (Eckstein et al., 2004; Lochmüller et al., 2002; Lochmüller et al., 2008) and its trabecular network (Arlot et al., 2008; Baum et al., 2013; Ding et al., 2002; Drews et al., 2008; Legrand et al., 2000; Müller et al., 2004; Pothuaud et al., 2002).

It should be noted that the purpose of the study was to advance scientific knowledge, not to develop the clinical management of antebrachial fractures.

When comparing the trabecular structure parameter BV/TV of the two animal species in the present study, it was found that the bone volume fraction of the dogs was lower compared to the cats. This result could favour an increased antebrachial fracture risk of dogs over cats. In the literature, it has been reported that BV/TV can partially predict the mechanical properties of a bone (Pothuaud et al., 2002). The lower the bone volume fraction, the higher the risk of fracture, because this bone parameter has a high correlation with the mechanical failure load of a bone (Arlot et al., 2008; Ding et al., 2002). Other canine microcomputed tomographic studies investigated trabecular bone parameters in the *Processus coronoideus medialis ulnae* (Fitzpatrick et al., 2016; Lau et al., 2013) to visualise changes in subchondral bone in MCD (medial coronoid disease)‐positive dogs or in the *Caput ossis femoris* (Scherzer et al., 2009) to identify changes in dogs with Legg Calve Perthes disease. Lau et al. (2013) found almost similar BV/TV values in the proximal ulna in both healthy and MCD dogs as in the present study. Scherzer et al. (2009) presented higher values of BV/TV at the femoral head of dogs compared to BV/TV of dogs in this study at the forearm. This deviation could be explained by the different location of the femoral head versus forearm bones and their different loads. With regard to cats, only one comparative study (Boyd et al., 2005) was found in the literature, which examined the changes in the feline proximal tibia after cranial cruciate ligament rupture using microcomputed tomography. Boyd et al. (2005) detected lower values for BV/TV compared to the results of the cats in the present study. The difference between the two studies within the cats could also be explained by the different measurement localisations of tibia versus forearm bones, as these bones are also exposed to different loads.

For the ratio of the trabecular surface area to the volume of trabecular bone (BS/BV) contradictory results compared to BV/TV were obtained. According to this, the values of dogs for BS/BV at all locations were mostly significantly higher than those of cats. This behaviour for trabecular surface density values can be explained by the other structural parameters. The high number of trabeculae in dogs resulted in a high value for BS/BV at low BV/TV (Scherzer et al., 2009).

In the present study, cats showed fewer trabeculae (Tb.N), mostly with a greater separation (Tb.Sp) than dogs; however, they were significantly thicker (Tb.Th). This could lead to the conclusion that cats have more stable forearm bones at the evaluated localisations than dogs with regard to trabecular thickness. According to the study by Ding et al. (2002), trabecular thickness also has a high correlation with the mechanical failure load and to existing osseous microdamage (Arlot et al., 2008). When comparing the values for Tb.Th at the radius and ulna of the dogs in the present study with the studies of Lau et al. (2013) and Fitzpatrick et al. (2016) at the *Processus coronoideus medialis ulnae* and Scherzer et al. (2009) at the *Caput ossis femoris*, they settled in comparable areas. For the structural parameter Tb.Sp in the present study, similar mean values were recorded for the dogs as in the elbow studies of Lau et al. (2013) and Fitzpatrick et al. (2016). Scherzer et al. (2009) determined lower values for Tb.Sp in the canine femur. This deviation could be explained by the different locations of the microcomputed tomographic evaluations and the different stress on the bones. A further reason for differing values could be the remodelling processes of the bones while aging. In the present study, mainly old dogs were analysed, whereas in the other studies young dogs between 15 weeks and 3 years were examined (Fitzpatrick et al., 2016; Lau et al., 2013; Scherzer et al., 2009). Body weight could also have an influence on the structural parameters, whereby in the present study only dogs below 10.0 kg were measured, while the other studies primarily included heavier dogs between 18 and 23 kg (Fitzpatrick et al., 2016; Lau et al., 2013; Scherzer et al., 2009). When comparing the feline results of radius and ulna of the present study with those from the feline tibia, Boyd et al. (2005) detected higher values for Tb.N and Tb.Sp and lower values for Tb.Th. Again, this could be explained by the different locations of the measurements and the load on the bones. In the study by Boyd et al. (2005) cats suffered from a cruciate ligament rupture with consequent bone pathology.

In the present study, higher connectivity was observed in the examined bones of dogs than those of cats. This could be due to the fact that dogs had more trabeculae, which were therefore better connected than the few thick trabeculae of cats.

Scherzer et al. (2009) reported similar results for Conn.D in their measurements in the canine femoral head compared to the results of the dogs in the radius and ulna in the present study. The connectivity of the feline tibia in the study by Boyd et al. (2005) was clearly higher than the results of the present study at the feline forearm, which again could be explained by the different locations of the tibia versus radius and ulna, the different load on the bones or the comparison of healthy cats versus cats with torn cruciate ligaments.

In the studies by Legrand et al. (2000), Ding et al. (2002) and Drews et al. (2008), the degree of anisotropy showed a negative correlation with trabecular bone fragility. In the present study, this value was significantly higher in cats than in dogs. This could therefore indicate for more stable forearm bones in cats. In the study by Hu et al. (2002), the anisotropy of dogs at lumbar vertebrae was comparable to the values of dogs at the radius and ulna in the present study. In the comparison within the cat studies, Boyd et al. (2005) found clearly higher values for anisotropy at the feline tibia than in the present study at the feline antebrachium. Again, the reason could be the different locations of the measurements, stress on the bones or osseous pathology.

In addition to trabecular bone parameters, diaphyseal cortical density (Mean Density) was also analysed in the present study, which was significantly higher in cats than in dogs. Thus, it can be assumed that the cortical diaphysis of the investigated antebrachial bones of the cats showed a higher stability.

In the literature, the prevalence of radius and ulna fractures in cats is described as 2.0–8.0% (Harari, 2002; Nolte et al., 2005), which is clearly lower than the prevalence in dogs at 18.0% (Boudrieau, 2003). The lower risk of fracture in cats could therefore be due to higher bone volume fraction (BV/TV), thicker trabeculae (Tb.Th), higher anisotropy (DA) and significantly higher diaphyseal cortical density (Mean Density).

In general, the differences in trabecular and cortical structure of the antebrachium between dogs and cats should not be ignored. Based on these results the cat should not be seen as a small dog.

## CONFLICT OF INTEREST

The authors declare that they have no conflict of interest.

## COMPLIANCE WITH ETHICAL STANDARDS

The experiment was approved by the Animal Ethics Committee of the Faculty of Veterinary Medicine, Ludwig‐Maximilians‐University, Munich, Germany.

### PEER REVIEW

The peer review history for this article is available at https://publons.com/publon/10.1002/vms3.619

## AUTHOR CONTRIBUTION

Franziska Planner: Conceptualisation, data curation, formal analysis, investigation, methodology, validation, visualisation, writing – original draft, writing – review & editing.

Franziska Feichtner: Conceptualisation, data curation, formal analysis, project administration, supervision, methodology, validation, visualisation, writing – original draft, writing – review & editing.

Andrea Meyer‐Lindenberg: Conceptualisation, project administration, resources, supervision, methodology, validation, visualisation, writing – original draft, writing – review & editing.

## Data Availability

The data that support the findings of this study are available from the corresponding author upon reasonable request.
